# Scale-Aware Compositional Inference Improves Reproducibility and Uncovers Convergent Aging Programs in Spatial Transcriptomics

**DOI:** 10.64898/2026.07.27.740958

**Published:** 2026-07-30

**Authors:** Deniz Parmaksiz, Steffy B. Manjila, Kyle C. McGovern, Donghui Shin, Ingvild E. Bjerke, Anirban Paul, Justin Silverman, Yongsoo Kim

## Abstract

Spatial transcriptomics enables analysis of molecular organization with anatomical context. Existing spatial differential expression methods are restricted to within-sample inference, forcing between-sample comparisons to rely on approaches adapted from single-cell RNA-seq. Here, we establish a scale-aware inference framework for spatial differential expression by modeling compositional constraints and variation in total RNA abundance rather than removing them through normalization, enabling calibrated between-sample inference at cell-level resolution. Our method produces more reliable results in simulated data and different spatial platforms. When applied to aged mouse brains, the analysis reveals converging aging-associated programs involving cellular signaling, membrane homeostasis, and neurovasculature across independent datasets.

## Full Text Availability

The license terms selected by the author(s) for this preprint version do not permit archiving in PMC. The full text is available from the preprint server.

